# A System of Human Anatomy, General and Special

**Published:** 1859-01

**Authors:** 


ART. VII.—*A System of Human Anatomy, General and Special.* By ERASMUS WILSON, F.R.S., author of "The Dissector's Manual," etc. etc. A new and improved American, from an enlarged London edition. Edited by WILLIAM H. GOBRECHT, M.D., Professor of Anatomy in the Philadelphia College of Medicine, etc. 8vo., pp. 616. Blanchard & Lea: Philadelphia, 1858.

THE very favorable reception which this work has met with since its first appearance renders any commendation at our hands superfluous, it having been used in every dissecting-room in this country, as a complete and faithful guide to the anatomical student, and as a book of reference to the more advanced physician. In the present edition the amount of matter has been extended and the number of wood-cuts largely increased. The mechanical execution is good, and the American editor has done his duty in reading proof-sheets and making a few additions, the prerogative, we believe, of editors of most of our reprints.

